# Gene expression profiling and protein–protein network analysis revealed prognostic hub biomarkers linking cancer risk in type 2 diabetic patients

**DOI:** 10.1038/s41598-023-49715-9

**Published:** 2023-12-18

**Authors:** Harshita Kasera, Rajveer Singh Shekhawat, Pankaj Yadav, Priyanka Singh

**Affiliations:** Department of Bioscience and Bioengineering, Indian Institute of Technology Jodhpur, NH 62, Nagaur Road, Karwar, Jodhpur, Rajasthan 342037 India

**Keywords:** Cancer, Systems biology, Genetic interaction

## Abstract

Type 2 diabetes mellitus (T2DM) and cancer are highly prevalent diseases imposing major health burden globally. Several epidemiological studies indicate increased susceptibility to cancer in T2DM patients. However, genetic factors linking T2DM with cancer have been poorly studied. In this study, we followed computational approaches using the raw gene expression data of peripheral blood mononuclear cells of T2DM and cancer patients available in the gene expression omnibus (GEO) database. Our analysis identified shared differentially expressed genes (DEGs) in T2DM and three common cancer types, namely, pancreatic cancer (PC), liver cancer (LC), and breast cancer (BC). The functional and pathway enrichment analysis of identified common DEGs highlighted the involvement of critical biological pathways, including cell cycle events, immune system processes, cell morphogenesis, gene expression, and metabolism. We retrieved the protein–protein interaction network for the top DEGs to deduce molecular-level interactions. The network analysis found 7, 6, and 5 common hub genes in T2DM vs. PC, T2DM vs. LC, and T2DM vs. BC comparisons, respectively. Overall, our analysis identified important genetic markers potentially able to predict the chances of PC, LC, and BC onset in T2DM patients.

## Introduction

Type 2 diabetes mellitus (T2DM) is a highly prevalent metabolic disorder that can occur at any age, albeit widespread in the middle (i.e., 45 years) to late age individuals. Insulin resistance, a condition in which muscle, liver, and fat cells fail to use insulin properly, precedes the onset of T2DM. Eventually, the beta cells of the pancreas cannot produce enough insulin due to progressive cell mass reduction or dysfunction. T2DM is characterized by insulin resistance and hyperglycemia^1^. The genome-wide association studies in the past have revealed some 403 distinct genetic variants in T2DM, which could influence beta-cell functioning, adipocytes, liver, skeletal muscle^2^, and many other tissues. As a result, it is not surprising that chronic T2DM can lead to additional complications such as nephropathy, cardiomyopathy, retinopathy, and neuropathy^3^. Consequentially, many differentially expressed genetic markers that could confer T2DM susceptibility were identified^4^. Subsequent bioinformatics analysis of these differentially expressed genes has revealed the genetic association of T2DM with these co-morbidities^5^. These findings have advanced our understanding of complications arising due to T2DM and have prospective applications in designing personalized prognostic and diagnostic tools for such heterogenic human diseases.

Cancer is another heterogenic disease that is also the second leading cause of human death^6^. It is characterized by unrestricted growth of abnormal cells. In some cases, these abnormal cells could metastasize to other parts of the human body. Liver, pancreatic, and breast cancers are among the most common cancer types^7^. It is well-known that T2DM and many common cancers share several risk factors like aging, obesity, and an unhealthy lifestyle^8^. Different epidemiological studies in the past suggest that T2DM condition increases the risk of several cancers, including liver, pancreatic^9^, breast^10,11^, and endometrial^12^. They report standardized incidence ratios to indicate an increased risk of cancers in T2DM patients. Pancreatic and liver cancers showed the highest standardized incidence ratios in different populations of T2DM patients from Denmark, Tyrol/Austria, Taiwan, Sweden, Australia, the Chinese mainland^13^, Finland^14^, and Lithuania. In addition, a few meta-analysis studies reported an increased risk of breast cancer in diabetic women^10,11^. There is no clear molecular understanding of T2DM link to specific cancer types yet. However, the state of insulin resistance, hyperinsulinemia, hyperglycemia, chronic inflammation, and increased oxidative stress in T2DM could probably elicit mitogenic pathways and cause these cancers^15^. Moreover, a few Mendelian randomization studies indicate a positive association between T2DM and the risk of pancreatic, breast, lung, liver, and kidney cancer^16,17^. Despite the availability of extensive evidence from epidemiological and meta-analysis that links cancer risk to T2DM, a systematic study of the shared genetic markers possibly predisposing this risk in T2DM patients is lacking for the common cancer types, namely pancreatic (PC), liver (LC), and breast (BC) cancer.

In this work, we performed gene expression analysis to identify predominant differential expressed genes (DEGs) from the peripheral blood mononuclear cell (PBMC) samples of T2DM patients, posing a risk towards three common cancer types (PC, LC, and BC). Their functional enrichment analysis indicated the involvement of gene expression, cell transport, and oxidation pathways. The protein–protein interaction (PPI) network provided common hub genes between T2DM and the three cancer types. We identified TGFB1 as a common hub gene between T2DM and PC/LC, significantly affecting survival in cancer patients. Therefore, the identified hub genes have a potential prognostic and therapeutic value in patients with T2DM patients and high cancer risks.

## Results

### Shared DEGs in T2DM and three common cancer types

**Figure 1:**
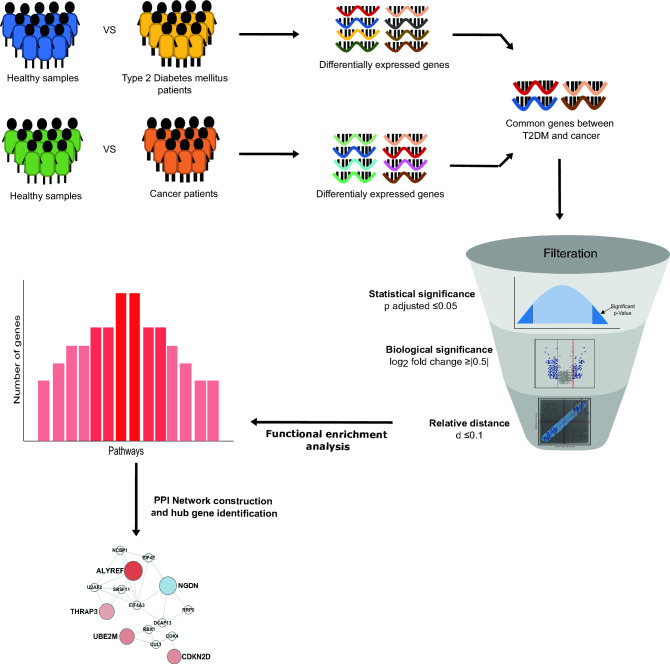
Schematic representation of the workflow used to identify differentially expressed genes (DEGs) and narrow down to the common hub genes in T2DM vs. respective cancers datasets.

Gene expression data of *Homo sapiens* in different diseased conditions were obtained from gene expression omnibus (GEO) database. Table 1 provides the summary of three different datasets used in our study. We employed a three-tiered filtering criterion to identify the shared DEGs between T2DM and three cancer types (Fig. 1). The raw gene expression datasets were normalized for each GEO study using the GEO2R tool (Supplementary Fig. S1). We identified 94 DEGs shared between T2DM and LC using our filtering criterion (Supplementary Fig. S2). Of these, 59 DEGs were upregulated, while 35 were downregulated (Supplementary Fig. S3). Likewise, for T2DM vs*.* BC comparison, we identified 16 shared DEGs (Supplementary Fig. S2), including 8 upregulated and 8 downregulated (Supplementary Fig. S3). For T2DM vs. PC, the three stringent filtering criteria resulted in an insignificant number of shared DEGs. However, using FDR ≤ 0.1 at filter 1, we identified 66 shared DEGs (Supplementary Fig. S2). Interestingly, the GSE15932 dataset also has data from 8 patients suffering from T2DM and PC disease. We applied the first two filtering criteria on T2DM & PC dataset, which identified 1203 DEGs (Supplementary Fig. S2). Surprisingly, we observed 69% (46 DEGs) shared DEGs of T2DM vs. PC overlapping with the DEG identified from T2DM & PC. For further functional analysis, we proceeded with the above 46 DEGs in T2DM vs. PC, where 26 were upregulated, and 20 were downregulated (Supplementary Fig S3). Similar validation could not be performed for T2DM vs. LC and T2DM vs. BC due to the unavailability of such a dataset. We observed a strong Pearson’s correlation (0.98 for T2DM vs. PC, 0.90 for T2DM vs. LC, and 0.87 for T2DM vs. BC) for the identified shared DEGs between T2DM and three common cancer types (i.e., PC, LC, and BC). Table 2 summarizes the number of shared DEGs narrowed down after applying our filtering criteria to each dataset.

**Table 1:** Overview of GEO datasets used in this study. The dash (–) symbol indicates that the information is not available.

Accession ID	Sample type	Sample size	Gender	Age (in years)	Country
GSE15932	T2DM	8	Male/female	43–80	China
	PC	8			
	T2DM & PC	8			
	Healthy	8			
GSE58208	LC	10	–	–	Singapore
	Healthy	5			
	Others	12			
GSE27562	BC	37	Female	–	USA
	Healthy	31			
	Others	94			

*T2DM:* type 2 diabetes mellitus, *PC:* pancreatic cancer, *LC:* liver cancer, *BC:* breast cancer.

**Table 2:** Summary of the number of genes filtered with three-tiered filtering criteria (statistical significance (filter 1; adjusted p-value ≤ 0.05 unless specified), biological significance (filter 2; log_2_FC ≥ |0.5|) and 10% relative distance around linear regression line of the correlated gene (filter 3).

Disease type	Number of genes			
	Common	Statistical significance	Biological significance	Relative distance
T2DM vs. PC	22,190	98 (adjusted p-value ≤ 0.1)	76	66 (46 common with T2DM & PC)
T2DM & PC	22,190	1996	1203	Not applicable
T2DM vs. LC	22,190	142	110	94
T2DM vs. BC	22,190	212	20	16

### Functional enrichment and pathway analysis of shared DEGs

To understand the functional relevance of identified common genes between T2DM and three cancers (PC, LC and BC), we utilized GO biological process and KEGG signaling pathway analysis tools from GENECODIS software. The GO biological process of the shared DEGs in T2DM vs. PC comparison showed enrichment of vesicle-mediated transport, protein export from the nucleus, engulfment of target by autophagosome, positive regulation of cellular protein metabolic process, positive regulation of protein localization to nucleus, etc. (Table 3). The KEGG pathway analysis results were insignificant for the T2DM vs. PC comparison. In the T2DM vs. LC comparison, the shared DEGs revealed enrichment in several critical biological processes such as ATP biosynthetic process, peptide modification, regulation of RNA splicing, regulated exocytosis, and neutrophil degranulation (Table 4). We also found enrichment of KEGG pathways shown in Supplementary Table S1, (section T2DM vs. LC), which includes synaptic vesicle cycle (hsa04721, p = 5.63 × 10^−2^), rheumatoid arthritis (hsa05323, p = 5.63 × 10^−2^), collecting duct acid secretion (hsa04966, p = 5.63 × 10^−2^), phagosome (hsa04145, p = 5.63 × 10^−2^), and oxidative phosphorylation (hsa00190, 9.05 × 10^−2^).

**Table 3:** Shows top significantly enriched GO:BP involving the identified DEGs for T2DM vs. PC patients.

Description	Annotation ID	Count	FDR	DEGs
Vesicle-mediated transport	GO:0016192	7	1.15 × 10^−3^	SFT2D1, TRAPPC1, RAB32, TFG, STXBP2, GSN, SYT15
Plus-end-directed vesicle transport along microtubule	GO:0072383	2	1.38 × 10^−2^	KIF13A, KIF3A
Receptor catabolic process	GO:0032801	2	3.52 × 10^−2^	TGFB1, SMURF1
Protein export from nucleus	GO:0006611	2	3.52 × 10^−2^	TGFB1, SMURF1
Engulfment of target by autophagosome	GO:0061736	1	3.52 × 10^−2^	SMURF1
Negative regulation of translation in response to endoplasmic reticulum stress	GO:1902010	1	3.52 × 10^−2^	SESN2
Response to muscle stretch	GO:0035994	2	3.52 × 10^−2^	GSN, NFKBIA
Septin ring assembly	GO:0000921	1	3.52 × 10^−2^	ANLN
Positive regulation of cellular protein metabolic process	GO:0032270	2	3.52 × 10^−2^	TGFB1, NFKBIA
Positive regulation of protein localization to nucleus	GO:1900182	2	3.60 × 10^−2^	SESN2, TGFB1

*FDR:* false discovery rate, *DEGs:* differentially expressed genes, *Count:* number of DEGs.

**Table 4:** Shows top significantly enriched GO:BP involving the identified DEGs for T2DM vs. LC comparison.

Description	Annotation ID	Count	FDR	DEGs
ATP biosynthetic process	GO:0006754	4	2.1 × 10^−3^	TGFB1, COX5B, ATP6V0C, ALDOA
Peptide modification	GO:0031179	2	1.58 × 10^−2^	GGT2, GGT1
Regulation of RNA splicing	GO:0043484	4	1.96 × 10^−2^	PTBP1, MBNL2, CDK11A, SRSF10
Negative regulation of mRNA splicing, via spliceosome	GO:0048025	3	1.96 × 10^−2^	PTBP1, SRSF7, SRSF10
Regulated exocytosis	GO:0045055	7	2.19 × 10^−2^	TGFB1, CTSD, CYBA, ATP6V0C, RHOG, ALDOA, DBNL
Neutrophil degranulation	GO:0043312	6	2.54 × 10^−2^	CTSD, CYBA, ATP6V0C, RHOG, ALDOA, DBNL
Regulation of mast cell degranulation	GO:0043304	2	9.16 × 10^−2^	UNC13D, STXBP2
Negative regulation of glomerular filtration by angiotensin	GO:0003106	1	9.16 × 10^−2^	CYBA
Reactive nitrogen species metabolic process	GO:2001057	1	9.16 × 10^−2^	PRDX5
Proton transmembrane transport	GO:1902600	4	9.16 × 10^−2^	ATP6V0E1, ATP6V0D1, ATP6V0C, COX5B

Similarly, analysis of common DEGs in T2DM vs. BC comparison led to the enrichment of hydrogen peroxide catabolic process, cellular oxidant detoxification, autophagic cell death, regulation of DNA recombination, hemoglobin biosynthetic process, and plasminogen activation from GO biological process (Table 5). The KEGG pathway analysis results were insignificant for the T2DM vs. BC comparison. Overall, cellular transport, gene expression, and cellular oxidation pathways were affected in this analysis, which motivated us further to perform the interaction analysis at the molecular level.

**Table 5:** Shows top significantly enriched GO:BP involving the identified DEGs for T2DM vs. BC comparison.

Description	Annotation ID	Count	FDR	DEGs
Hydrogen peroxide catabolic process	GO:0042744	3	1.10 × 10^−4^	HBD, HBG2, HBG1
Cellular oxidant detoxification	GO:0098869	3	1.85 × 10^−3^	HBD, HBG2, HBG1
Autophagic cell death	GO:0048102	1	5.40 × 10^−2^	CDKN2D
Regulation of DNA recombination	GO:0000018	1	5.40 × 10^−2^	ALYREF
Hemoglobin biosynthetic process	GO:0042541	1	5.40 × 10^−2^	ALAS2
Plasminogen activation	GO:0031639	1	5.40 × 10^−2^	PGK1
Positive regulation of circadian rhythm	GO:0042753	1	5.40 × 10^−2^	THRAP3
DNA synthesis involved in DNA repair	GO:0000731	1	6.05 × 10^−2^	CDKN2D
Protein neddylation	GO:0045116	1	6.05 × 10^−2^	UBE2M
Maturation of SSU-rRNA from tricistronic rRNA transcript (SSU-rRNA, 5.8S rRNA, LSU-rRNA)	GO:0000462	1	6.96 × 10^−2^	NGDN

### Deducing molecular level interactions using PPI network

**Figure 2:**
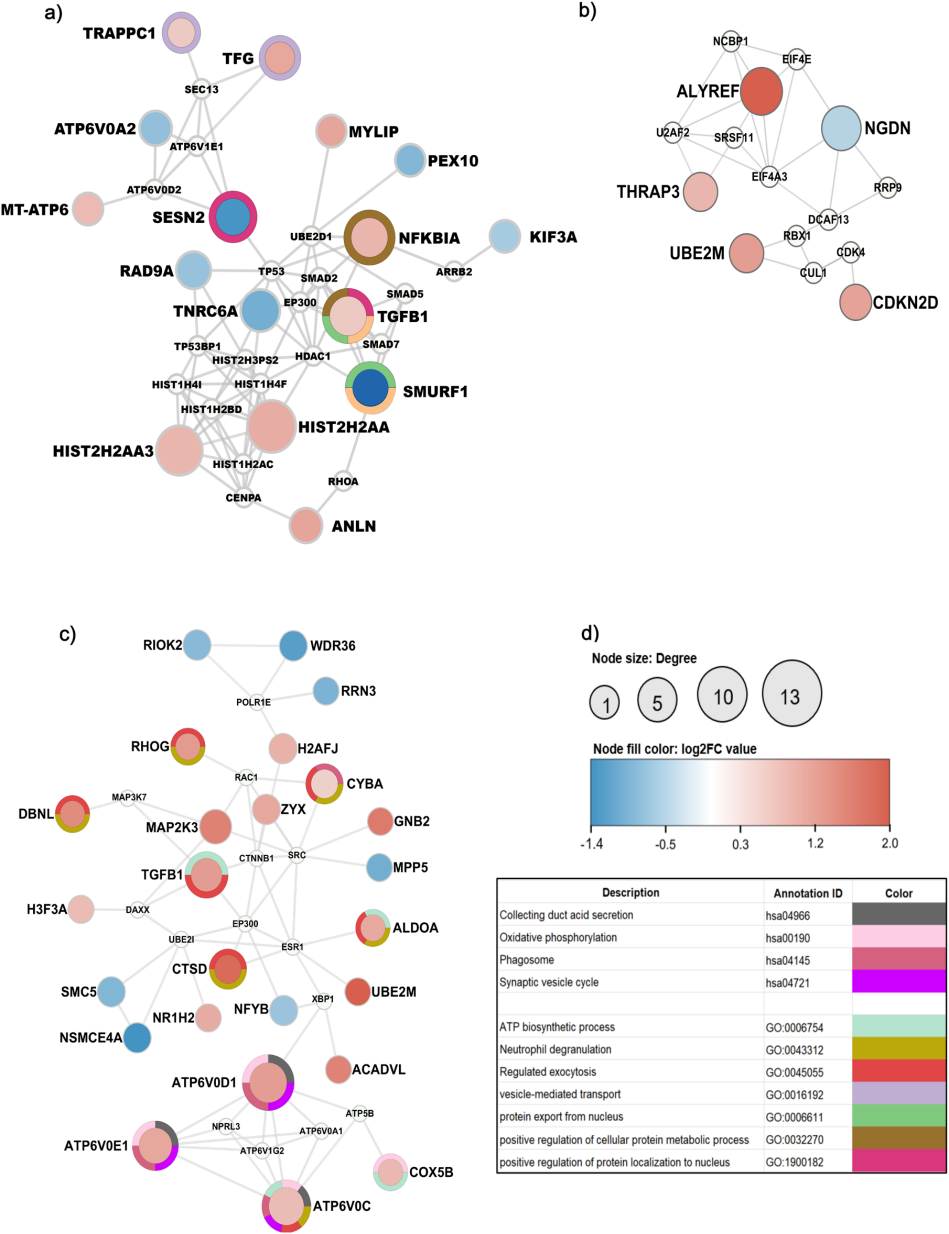
Protein–protein interaction network for (**a**) T2DM vs. PC, (**b**) T2DM vs. BC, and (**c**) T2DM vs. LC. The colored circle around the nodes represents different enriched pathways to which these nodes are linked. The small nodes (in white color) indicate additional interactors. The node color indicates overexpressed genes (red) and under-expressed genes (blue). The node size represents the node’s degree, and the node’s color intensity represents the log_2_ fold change (FC) value of differentially expressed genes (DEGs). The color chart (**d**) illustrates different enriched pathways and their annotation identity.

The above analysis linked identified common genes to specific biological pathways, which intrigued us to investigate their relationship at the molecular level. We constructed the protein–protein interaction (PPI) networks of identified common DEGs between T2DM and three cancer types (PC, LC, and BC). Our analysis yielded 16 nodes (genes) in T2DM vs. PC, 25 in T2DM vs. LC, and 5 in T2DM vs. BC in the main connected PPI networks (Supplementary Table S2, Fig. 2a–c).

In the PPI network analysis, the node size reflects their degree, and node color indicates the expression pattern, thus making it possible to deduce certain hub genes for respective disease conditions. Moreover, network analysis provides information on the important hub genes and enriched processes and pathways involving major hub nodes as represented by a colored ring around each node (Fig. 2a–d). Our network analysis revealed important genes associated with more than one biological pathway, thus suggesting their evident involvement in the respective disease conditions. The important genes from the T2DM vs. PC network included, *HIST2H2AA3*, *HIST2H2AA4*, *NFKBIA*, *SESN2*, *SMURF1*, *TGFB1*, *TNRC6A* (Fig. 2a). The T2DM vs. BC network could not reveal the genes associated with the biological pathways due to insufficient DEGs (Fig. 2b). For the T2DM vs. LC network, *ALDOA, ATP6V0D1, ATP6V0C, ATP6V0E1, TGFB1, CYBA, CTSD, and DBNL* genes were considered significant (Fig. 2c).

Interestingly, a common gene, *TGFB1,* between T2DM vs. PC and T2DM vs. LC, is upregulated in both conditions. This gene codes for a growth factor in cell proliferation, differentiation, and death. We used these findings as a basis for further investigation of hub genes.

### Hub genes identification and survival analysis

**Figure 3:**
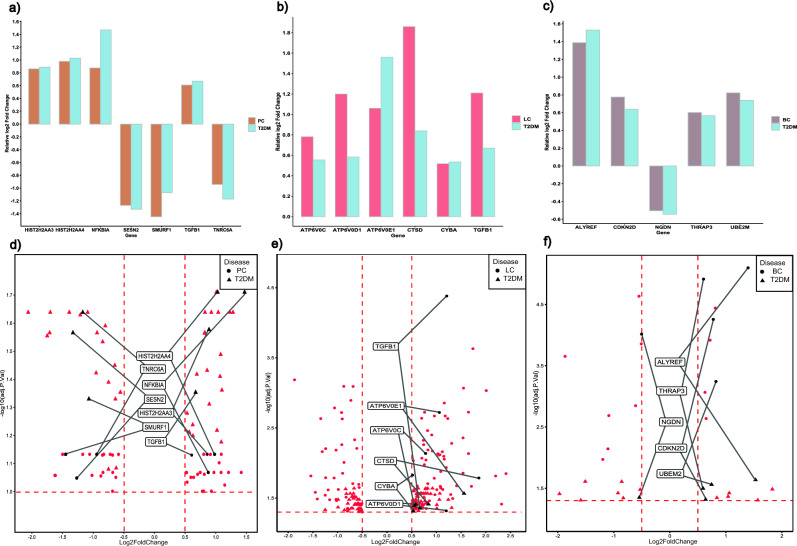
The barplot (**a**–**c**) shows log_2_ fold change expression of identified hub genes in T2DM vs. PC, T2DM vs. LC, and T2DM vs. BC comparison, respectively. The volcano plots (**d**–**f**) highlight the common hub genes for T2DM vs. PC, T2DM vs. LC, and T2DM vs. BC, respectively. The symbols represent whether the p-value and log_2_FC value of that particular DEG is for T2DM (triangle) or three cancers (circle). The shared hub genes are labeled in black, while other DEGs are shown in lighter colors.

We ranked the nodes in our PPI network analysis based on eleven different topological features using the cytoHubba plugin in the Cytoscape tool (see details in the materials and methods section). Thus, we identified the top 15 genes for T2DM vs. PC and T2DM vs. LC (Supplementary Table S3). We noticed several hub genes such as *HIST2H2AA4*, *SESN2*, and *TNRC6A* for T2DM vs. PC and *ATP6V0D1*, *ATP6V0C* and *TGFB1* for T2DM vs. LC that were top-ranked in almost all the computed topological features. This analysis was not performed for T2DM vs. BC due to the insufficient number of identified common DEGs. We further narrowed common hub genes based on their top ranking and commonness across the computed topological features. Accordingly, we could identify seven genes as the hub genes for the T2DM vs. PC comparison. The relative log_2_ fold expression of these genes in two diseased conditions is represented in Fig. 3a. For the T2DM vs. LC comparison, we identified six hub genes. We found similar expression levels of these genes in T2DM and LC disease conditions, as indicated in Fig. 3b. For the T2DM vs. BC comparison, five genes identified from PPI network analysis were considered the hub genes, as represented in Fig. 3c. We identified 17 hub genes combined for the three comparisons, which could serve as potential biomarkers. Further, survival analysis was also performed for all the common hub genes using a web resource UALCAN, which analyzes publicly available cancer OMICS data^18,19^. The survival analysis revealed 4 hub genes (*ATP6V0C*, p < 0.051; *ATP6V0D1*, p < 0.02; *ATP6V0E1*, p < 0.0002 and *TGFB1*, p = 0.042) with significant p-value ≤ 0.05 to be linked with poor survival (Supplementary Fig. S4). The expression levels of these four hub genes were similar to our analysis. Interestingly, we found a common hub gene *TGFB1* between T2DM vs. pancreatic cancer and T2DM vs. liver cancer patients, which significantly affects survival. In the case of breast cancer samples, the diagnosis was benign, so, likely, the survival is not dependent on the identified common hub signatures for these samples.

We further validated the identified prognostic 17 hub markers from our study using three additional publicly available datasets for T2DM patients (Supplementary Fig. S5a). Our validation analysis revealed overlapping functionally-enriched gene ontology biological processes (Supplementary Fig. S5b–e). Out of the 17 hub genes, we could validate 12 genes (p-value ≤ 0.05) based on their expression profile (Supplementary Fig. S5f), despite differences in their sample sources (whole blood sample vs. PBMC) and variability in detection ranges due to different platforms (Supplementary Fig. S6).

## Discussion

T2DM and cancer have burdened the health sector throughout the world. Recently, several epidemiological studies have indicated a causal link between T2DM and common cancer types. However, the genetic association of T2DM to these cancers remains largely unknown. Our work identifies a genetic association between T2DM and three common cancer types, i.e., PC, LC, and BC. Our analysis identified 7, 6, and 5 hub genes showing a correlation between T2DM and the three cancers, i.e., PC, LC, and BC, respectively.

The KEGG pathway and GO biological process analyses showed enrichment of vesicle-mediated transport, vital in tumor microenvironment remodeling and transport of secretory insulin or other circulating mediators in diabetes^20–23^. Neutrophil deregulation is known to be associated with diabetes^24^ and cancer cell progression, metastasis, and activating dormant cancer cell^25^. Moreover, we noticed positive regulation of metabolic and cellular protein metabolic processes in T2DM vs. PC conditions. Our analysis also revealed important processes contributing to gene expression, including regulation of RNA splicing and protein degradation, which are required to maintain homeostasis. In nutshell, we identified seven hub genes (*HIST2H2AA3, HIST2H2AA4, NFKBIA, SESN2, SMURF1, TGFβ1, TNRC6A*) in T2DM vs. PC, six common hub genes (*ATP6V0D1, ATP6V0C, ATP6V0E1, CTSD, CYBA, TGFβ1*) in T2DM vs. LC and five common hub genes (*ALYREF*, *CDKN2D*, *NGDN*, *THRAP3*, *UBE2M*) in T2DM vs. BC, respectively (Fig. 3). Supplementary Table S4 provides the detailed function and description of these hub genes. Noteworthy, The *NFKBIA* gene involved in the NFKB pathway has a crucial role in the initiation and progression of PC^25^, and its gene polymorphism has an implicit role in the prognosis of T2DM^26^. *TGFB1* is involved at an early and advanced stage in liver tumorigenesis^27^, and TGF-β signaling plays diverse roles in β cell development and functioning that has an impactful role in diabetic condition^28^. The V-ATPase H+ transporting genes (*ATP6V0D1, ATP6V0C, ATP6V0E1*) maintain the intracellular pH and thus are critical for the Warburg effect observed in the cancer cells^29^. The *CTSD* gene has a role in decreasing the expression of IGFBP3, contributing to mitogenesis in hepatoma cells^30^, and it also has increased plasma activity in T2DM male patients^31^. The *ALYREF* gene plays a significant role in cellular growth, apoptosis, and mitochondrial energy metabolism in BC^32^, and as a 5mC-related gene that could have a functional role in T2DM^33^. THRAP3 deficiency sensitizes BC cells, suggesting a probable involvement in DDR^34^. Also, THRAP3 plays a direct role in controlling diabetic gene programming by interacting with PPARγ^35^.

Thus, our analysis provides insight into biological and molecular events that could link T2DM with three common cancer types (PC, LC, and BC). Further, the identified genetic markers hold the potential to predict the chances of cancer onset in T2DM patients. However, systematic approaches for data collection, which consider variations in genetic profiling based on ethnicity, sex, and age, could further expand our understanding. Notably, such markers in T2DM patient PBMC samples predisposing to increased cancer risk could help diagnosis at an early stage and provide benefits for developing personalized therapeutic strategies.

## Material and methods

### Microarray data collection

The raw gene expression data of *Homo sapiens* used in this study was available at the gene expression omnibus (GEO; http://www.ncbi.nlm.nih.gov/geo/) database. The studies used in this work specifically comprised the expression profile of the peripheral blood mononuclear cells (PBMCs) using the Affymetrix platform GPL570. The GSE15932 dataset included expression data of 32 PBMC samples containing 8 healthy individuals, 8 T2DM, 8 PC, and 8 samples of both T2DM and PC patients. The LC gene expression data along with their respective healthy controls were collected from GSE58208. The study comprised of gene expression analysis of PBMC samples from healthy individuals, liver cancer and hepatitis B carrier patients. The GSE27562 datasets collected the breast cancer gene expression profile and the healthy women samples. Blood was collected from 37 women who have benign breast cancer in comparison to 31 healthy individuals.

### Data pre-processing and identification of DEGs

The raw gene expression data was normalized using the GEO2R tool. The prospective shared genetic markers between T2DM and three cancer types (PC, LC, and BC) were obtained by applying three filters. Our first filter is based on the adjusted p-value, indicating the statistical significance of differentially expressed genes. We considered genes having adjusted p-value ≤ 0.05 for further analysis. Our second filtering criteria is based on the relative log_2_FC of gene expression, calculated for disease condition samples with respect to healthy condition samples indicating the biologically significant genes. We retained genes having absolute log_2_FC ≥ |0.5| for further analysis. Lastly, we kept correlated genes falling within the 10% interval from a regression line passing through the origin (i.e., x = 0 and y = 0 on an xy plane) between log_2_FC in T2DM and respective cancer types. Only upregulated or downregulated genes in both conditions were selected for further analysis. Pearson correlation was calculated for the genes narrowed down after applying filtering criteria in each condition, using the cor function in the R software.

### Functional enrichment analysis of DEGs

The gene ontology (GO) enrichment analysis and Kyoto Encyclopedia of Genes and Genomes (KEGG) pathway analysis were performed to annotate the biological function of the DEGs using the online software GENECODIS. We considered a cut-off of the false discovery rate (FDR) at 0.10 to define the significance level.

### Protein–protein interaction (PPI) network construction and visualization

The PPI network analysis was performed based on the Search Tool for the Retrieval of Interacting Genes (STRING, https://string-db.org), a database of known and predicted protein–protein interactions. We used genes differentially expressed in T2DM and three cancer types (PC, LC, and BC) to construct the PPI network. Interaction with a score > 0.8 was deemed statistically significant. The PPI network was created using Cytoscape (version 3.8.2), an open-source software for visualizing molecular interaction networks and biological pathways.

### Hub genes identification

The hub genes were explored using the cytoHubba application in the Cytoscape tool. For this purpose, the PPI network was analyzed to compute various topological features, including degree, maximal clique, centrality, density of maximum neighborhood component, maximum neighborhood component, edge percolated component, bottleneck, eccentricity, closeness, radiality, betweenness, and stress. The top 15 nodes were considered notable genes in the network for each computed topological feature. The nodes common to all topological features were regarded as the critical hub genes or key nodes in the network.

### Survival analysis

The survival analysis was performed using UALCAN to analyze expression data from publicly available databases (http://ualcan.path.uab.edu/index.html)^18,19^. The correlation between hub gene expression and survival in the three cancer types was analyzed by UALCAN. The patients with cancer were split into two groups according to the expression of a particular gene (high vs. low/medium expression), and the survival time was compared between the two groups.

### Validation dataset analysis

To validate the discovery dataset (GSE15932), we shortlisted 3 validation datasets (GSE23561, GSE69528, and GSE189005) matching criteria of T2DM, *Homo Sapien* taxid, expression profile by array, and blood samples. Raw gene expression data for GSE69528 and GSE189005 datasets were normalized using GEO2R tool. GSE23561 raw gene expression data was normalized by "normalizeBetweenArrays" command of Bioconductor package limma V3.562 in R4.3.0. Significant DEGs in T2DM were obtained after applying a filter for adjusted p-value ≤ 0.05 and log2FC ≥ |0.5|. Functional enrichment analysis of the obtained DEGs was performed using GENECODIS. For representation, only functionally enriched GO:BP overlapping with the discovery datasets were shown in Fig S5c–e. The expression profile of the 17 hub genes among discovery and validation datasets were plotted and color-coded (yellow and green shaded boxes) when found significant (p ≤ 0.05) in any of the validation datasets.

### Ethical standards

The manuscript does not contain clinical studies or patient data.

### Supplementary Information

Supplementary Information.

## Data Availability

The datasets used during the current study are available on the gene expression omnibus database (URL: http://www.ncbi.nlm.nih.gov/geo/). The datasets used in this study are available with the following accession IDs: GSE15932, GSE58208 and GSE27562. The codes used in the study are available on GitHub (https://github.com/RajveerSingh27R/Cross-Phenotype-Analysis).

## Abbreviations

T2DM: Type 2 diabetes mellitus
PC: Pancreatic cancer
LC: Liver cancer
BC: Breast cancer
DEGs: Differentially expressed genes
PPI: Protein-protein interaction
PBMC: Peripheral blood mononuclear cells
log_2_FC: Log_2_ fold change
GO: Gene ontology
KEGG: Kyoto encyclopedia of genes and genomes
FDR: False discovery rate

## References

[1] DeFronzo RA (2015). Type 2 diabetes mellitus. Nat. Rev. Dis. Primers.

[2] Mahajan A (2018). Fine-mapping type 2 diabetes loci to single-variant resolution using high-density imputation and islet-specific epigenome maps. Nat. Genet..

[3] Daryabor G, Atashzar MR, Kabelitz D, Meri S, Kalantar K (2020). The Effects of type 2 diabetes mellitus on organ metabolism and the immune system. Front. Immunol..

[4] Morris AP (2012). Large-scale association analysis provides insights into the genetic architecture and pathophysiology of type 2 diabetes. Nat. Genet..

[5] Azzam SK (2019). Genetic associations with diabetic retinopathy and coronary artery disease in emirati patients with type-2 diabetes mellitus. Front. Endocrinol. (Lausanne)..

[6] Rositch AF (2020). Global burden of cancer attributable to infections: The critical role of implementation science. Lancet Glob. Health.

[7] Siegel RL, Miller KD, Jemal A (2016). Cancer statistics, 2016. CA Cancer J..

[8] Giovannucci E (2010). Diabetes and cancer: A consensus report. Diabetes Care.

[9] Pan X-F (2018). Type 2 diabetes and risk of incident cancer in China: A prospective study among 0.5 million chinese adults. Am. J. Epidemiol..

[10] Boyle P (2012). Diabetes and breast cancer risk: A meta-analysis. Br. J. Cancer.

[11] Hardefeldt PJ, Edirimanne S, Eslick GD (2012). Diabetes increases the risk of breast cancer: A meta-analysis. Endocr.-Related Cancer.

[12] Saed L (2019). The effect of diabetes on the risk of endometrial Cancer: An updated a systematic review and meta-analysis. BMC Cancer.

[13] Wang M (2015). Cancer risk among patients with type 2 diabetes mellitus: A population-based prospective study in China. Sci. Rep..

[14] Saarela K (2019). Cancer incidence among Finnish people with type 2 diabetes during 1989–2014. Eur. J. Epidemiol..

[15] Shlomai G, Neel B, LeRoith D, Gallagher EJ (2016). Type 2 diabetes mellitus and cancer: The role of pharmacotherapy. J. Clin. Oncol..

[16] Pearson-Stuttard J (2021). Type 2 diabetes and cancer: An umbrella review of observational and mendelian randomization studies. Cancer Epidemiol. Biomark. Prev..

[17] Shen B (2022). Association between age at diabetes onset or diabetes duration and subsequent risk of pancreatic cancer: Results from a longitudinal cohort and mendelian randomization study. Lancet Reg. Health West Pac..

[18] Chandrashekar DS (2022). UALCAN: An update to the integrated cancer data analysis platform. Neoplasia.

[19] Chandrashekar DS (2017). UALCAN: A portal for facilitating tumor subgroup gene expression and survival analyses. Neoplasia.

[20] Bracey, K. M., Gu, G. & Kaverina, I. Microtubules in pancreatic β cells: Convoluted roadways toward precision. *Front. Cell Develop. Biol*. **10**, 915206 (2022). 10.3389/fcell.2022.915206 PMC9305484 35874834

[21] Wattanathamsan, O. & Pongrakhananon, V. Emerging role of microtubule-associated proteins on cancer metastasis. *Front. Pharmacol*. **13**, 935493 (2022). 10.3389/fphar.2022.935493 PMC9515585 36188577

[22] NorenHooten N, Evans MK (2020). Extracellular vesicles as signaling mediators in type 2 diabetes mellitus. Am. J. Physiol. Cell Physiol..

[23] Li Y, Zhao W, Wang Y, Wang H, Liu S (2022). Extracellular vesicle-mediated crosstalk between pancreatic cancer and stromal cells in the tumor microenvironment. J. Nanobiotechnol..

[24] Wong SL (2015). Diabetes primes neutrophils to undergo NETosis, which impairs wound healing. Nat. Med..

[25] Silke J, O’Reilly LA (2021). NF-κB and pancreatic cancer; chapter and verse. Cancers (Basel).

[26] Raza W, Ghafoor S, Abbas SZ, Muhammad SA (2020). Polymorphic evaluation of NFKBIA and SRR with type 2 diabetes mellitus in population of southern Punjab. Meta Gene.

[27] Tu S, Huang W, Huang C, Luo Z, Yan X (2019). Contextual regulation of TGF-β signaling in liver cancer. Cells.

[28] Wang H-L, Wang L, Zhao C-Y, Lan H-Y (2022). Role of TGF-beta signaling in beta cell proliferation and function in diabetes. Biomolecules.

[29] Sun H, Chen L, Cao S, Liang Y, Xu Y (2019). Warburg effects in cancer and normal proliferating cells: Two tales of the same name. Genom. Proteom. Bioinform..

[30] Ruiz-Blázquez P, Pistorio V, Fernández-Fernández M, Moles A (2021). The multifaceted role of cathepsins in liver disease. J. Hepatol..

[31] Ding, L. *et al.* Plasma cathepsin D activity rather than levels correlates with metabolic parameters of type 2 diabetes in male individuals. *Front. Endocrinol*. **11**, 575070 (2020). 10.3389/fendo.2020.575070 PMC7554511 33101209

[32] Klec C (2022). ALYREF, a novel factor involved in breast carcinogenesis, acts through transcriptional and post-transcriptional mechanisms selectively regulating the short NEAT1 isoform. Cell Mol. Life Sci..

[33] Song, Y. *et al.* Comprehensive analysis of key m5C modification-related genes in type 2 diabetes. *Front. Genet*. **13**, 1015879 (2022). 10.3389/fgene.2022.1015879 PMC9582283 36276976

[34] Beli P (2012). Proteomic investigations reveal a role for RNA processing factor THRAP3 in the DNA damage response. Mol. Cell.

[35] Choi JH (2014). Thrap3 docks on phosphoserine 273 of PPARγ and controls diabetic gene programming. Genes Dev..

