# Supplementary material for: Gene expression profiling and protein–protein network analysis revealed prognostic hub biomarkers linking cancer risk in type 2 diabetic patients
Source: Sci Rep. 2023 Dec 18;13:22605. doi: 10.1038/s41598-023-49715-9 (PMC10730526; doi:10.1038/s41598-023-49715-9)
Supplement: Supplementary file 1 — Supplementary Information. [file 41598_2023_49715_MOESM1_ESM.docx]

# SUPPLEMENTARY DATA

**Gene expression profiling and protein-protein network analysis revealed prognostic hub biomarkers linking cancer risk in type 2 diabetic patients**

Harshita Kasera^1#^, Rajveer Singh Shekhawat^1#^, Pankaj Yadav^1*^ and Priyanka Singh^1*^

^1^Department of Bioscience & Bioengineering, Indian Institute of Technology Jodhpur

NH 62, Nagaur Road, Karwar 342037, Jodhpur, Rajasthan, India.

# Equal contribution

^*^Correspondence to: [priyankasingh@iitj.ac.in](mailto:priyankasingh@iitj.ac.in); [pyadav@iitj.ac.in](mailto:pyadav@iitj.ac.in)


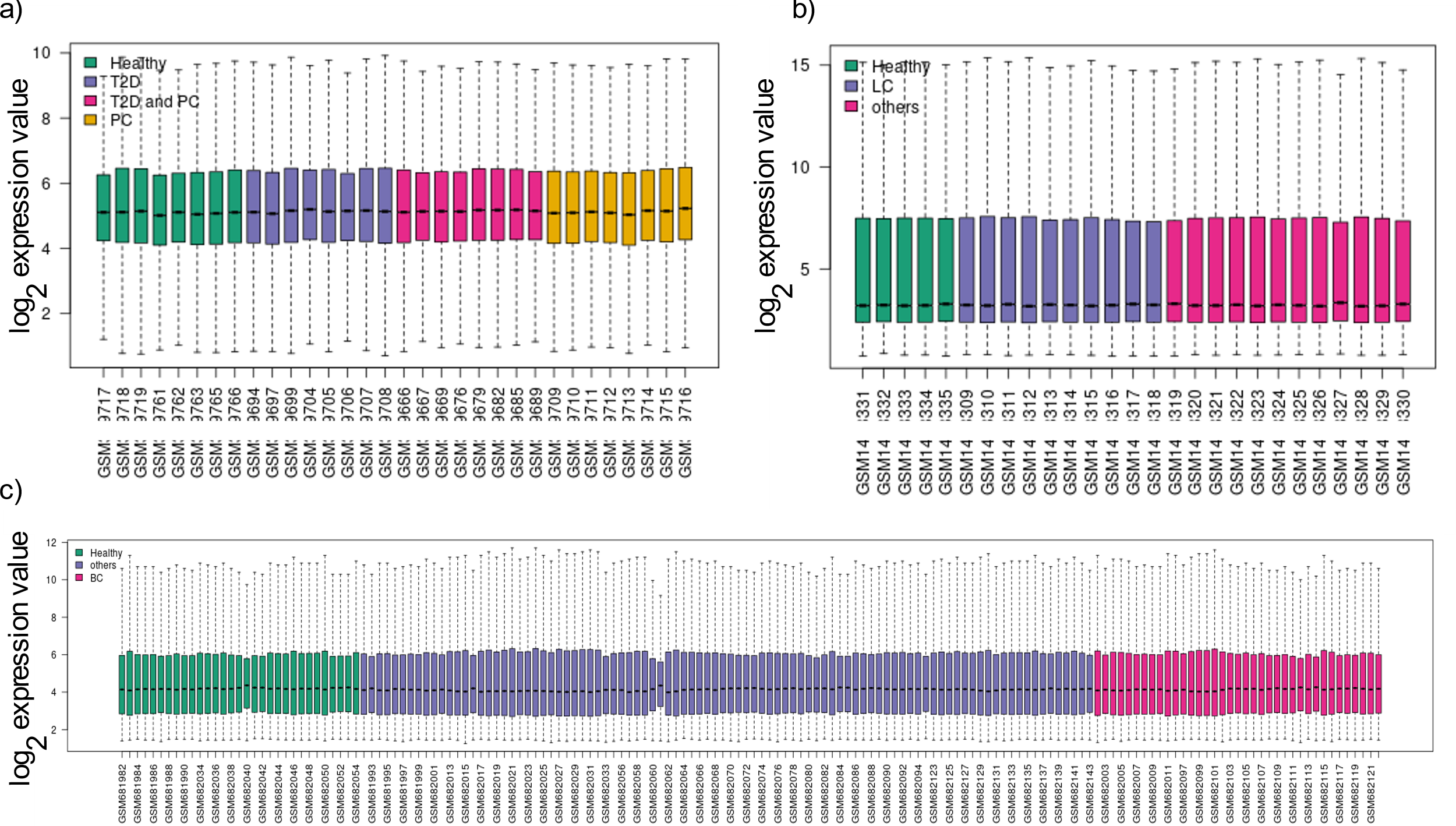


**Figure S1**. Boxplot of RMA (robust multichip average) normalized gene expression datasets: **(a)** GSE15932 study having patients suffering from only type 2 diabetes mellitus (T2DM), only pancreatic cancer (PC), both T2DM and PC and healthy samples, **(b)** GSE58208 study having patients suffering from liver cancer (LC), healthy samples and others. **(c)** GSE27562 study having patients suffering from breast cancer (BC), healthy samples and others.


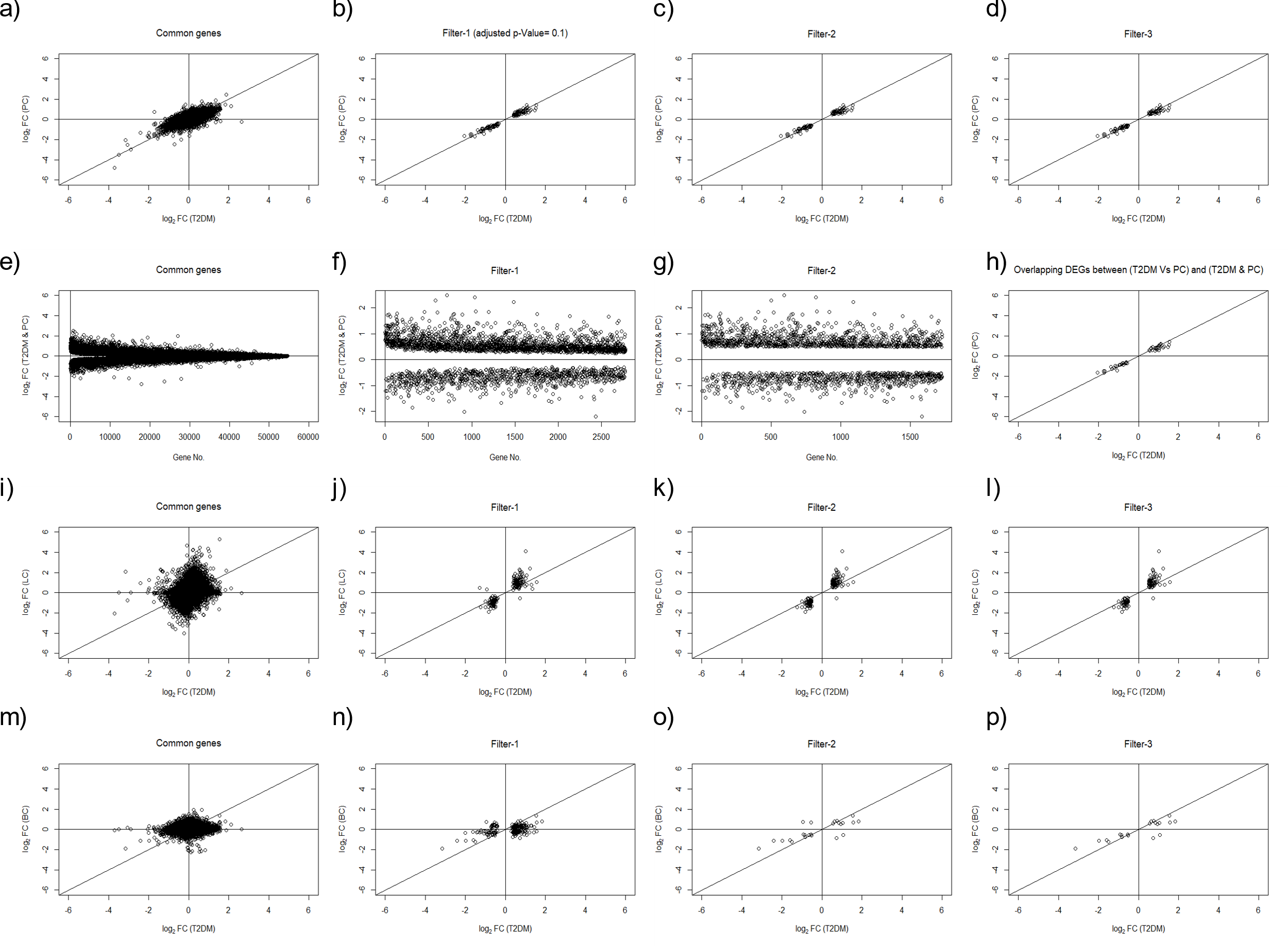


**Figure** **S2**. Scatter plot showing filtration of genes between T2DM and three cancer types (PC, LC, BC). **(a-d)** T2DM *vs* PC. Initially, expression data on 22190 common genes was available (a). p.adjusted≤ 0.1 in filter 1 followed by next two filters resulted in narrowed down 66 genes. **(e-g)** T2DM and PC. Initially 22190 genes were obtained from patients suffering from both T2DM and PC. **(h)** 46 DEGs common in T2DM vs PC (d) and T2DM & PC (g) were used for further analysis. **(i-l)** T2DM *vs* LC. Initially, expression data on 22190 common genes was available. 95 genes were obtained after the filtering criterion. **(m-p)** T2DM *vs* breast cancer (BC). 22190 genes expression data was provided in common and 17 genes were obtained after the filtering criterion


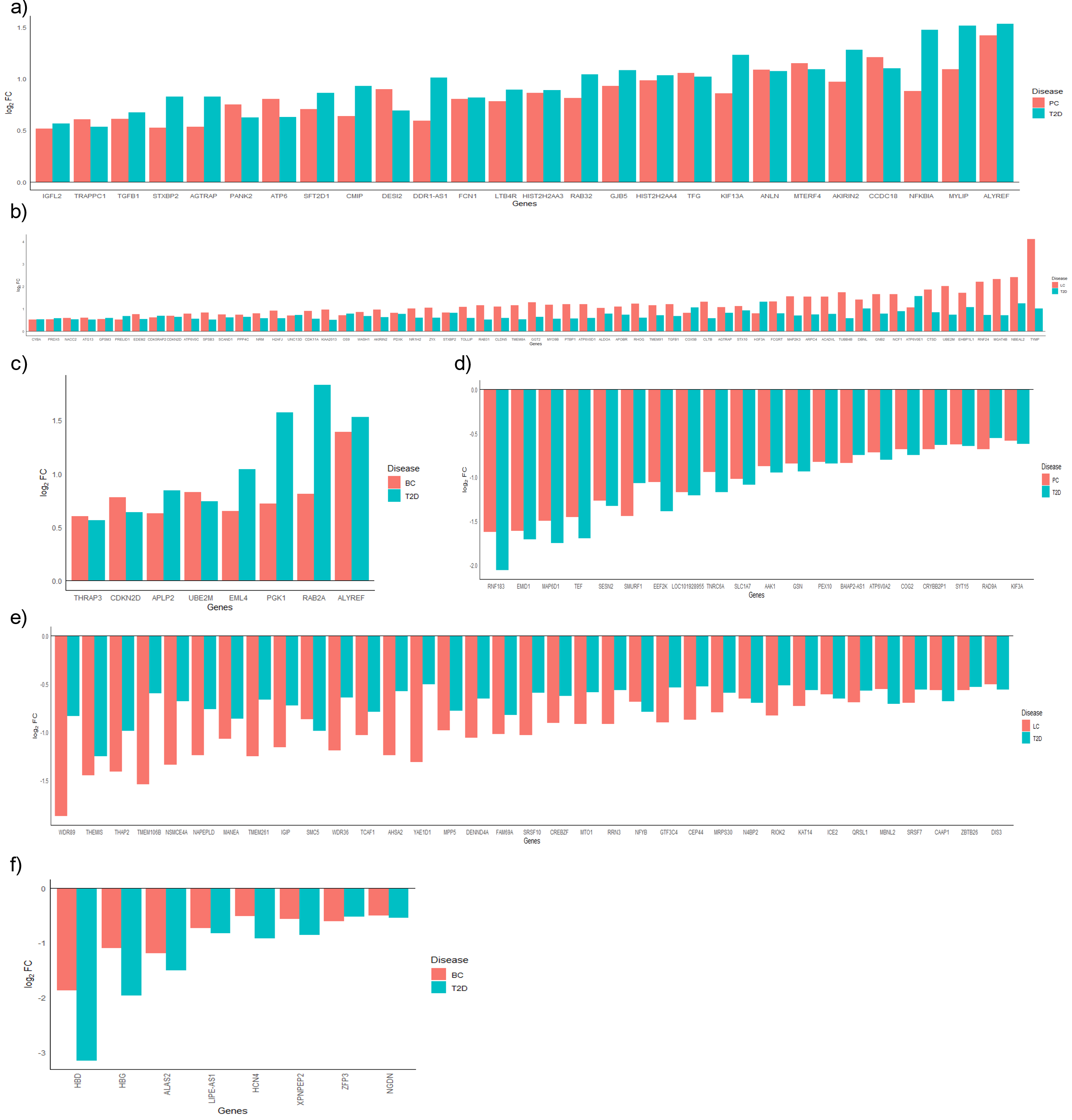


**Figure S3.** Expression pattern of the genes obtained after filtering in T2DM and cancer types. Upregulated genes having log_2_ fold ≥ 0.5 **(a)** T2DM *vs* PC **(b)** T2DM *vs* LC **(c)** T2DM *vs* BC. Downregulated genes having log_2_ fold ≤ -0.5 **(d)** T2DM *vs* PC, **(e)** T2DM *vs* LC, **(f)** T2DM *vs* BC


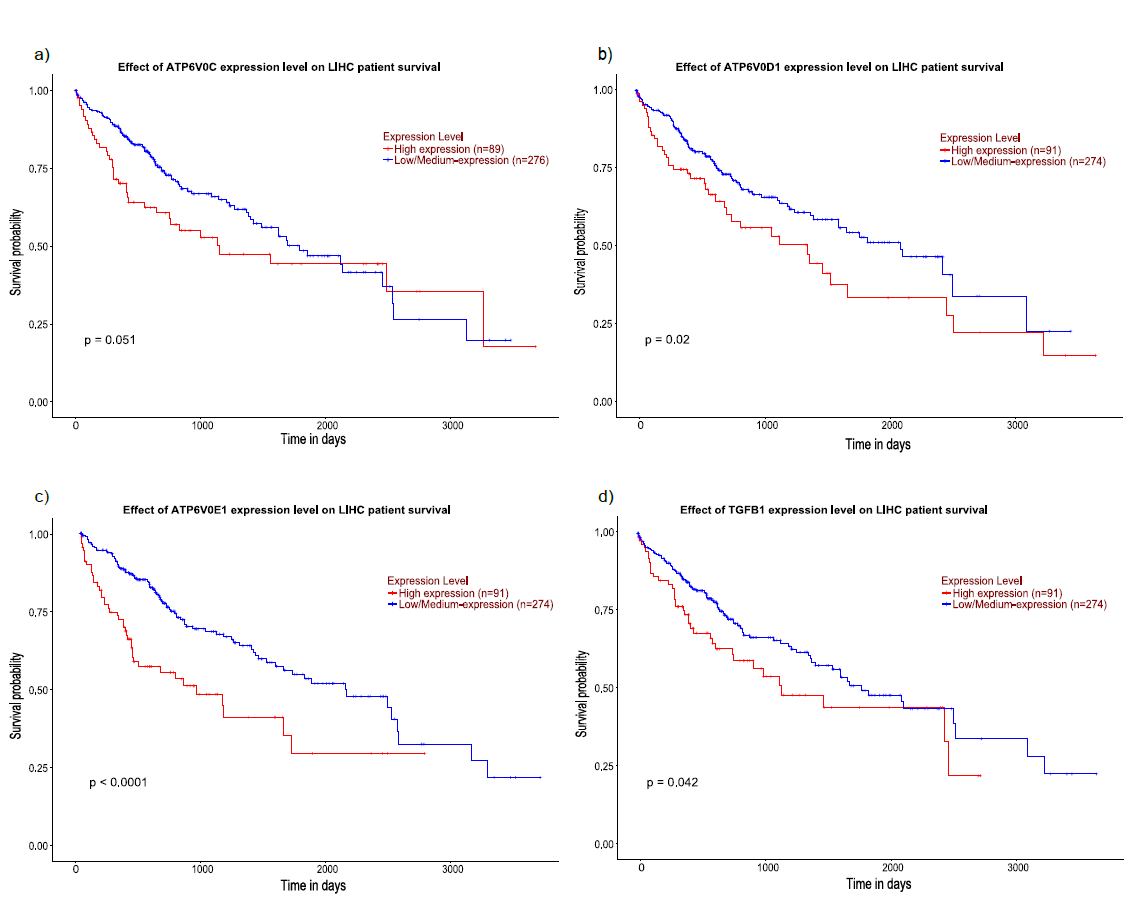


**Figure S4.** Kaplan-Meier plots showing the association of hub genes expression levels with patient survival. The survival analysis results showed the common hub genes linked with poor survival as *ATP6V0C* (p<0.051), *ATP6V0D1* (p<0.02), *ATP6V0E1* (p<0.0002) and *TGFB1* (p=0.042) in liver cancer, respectively.


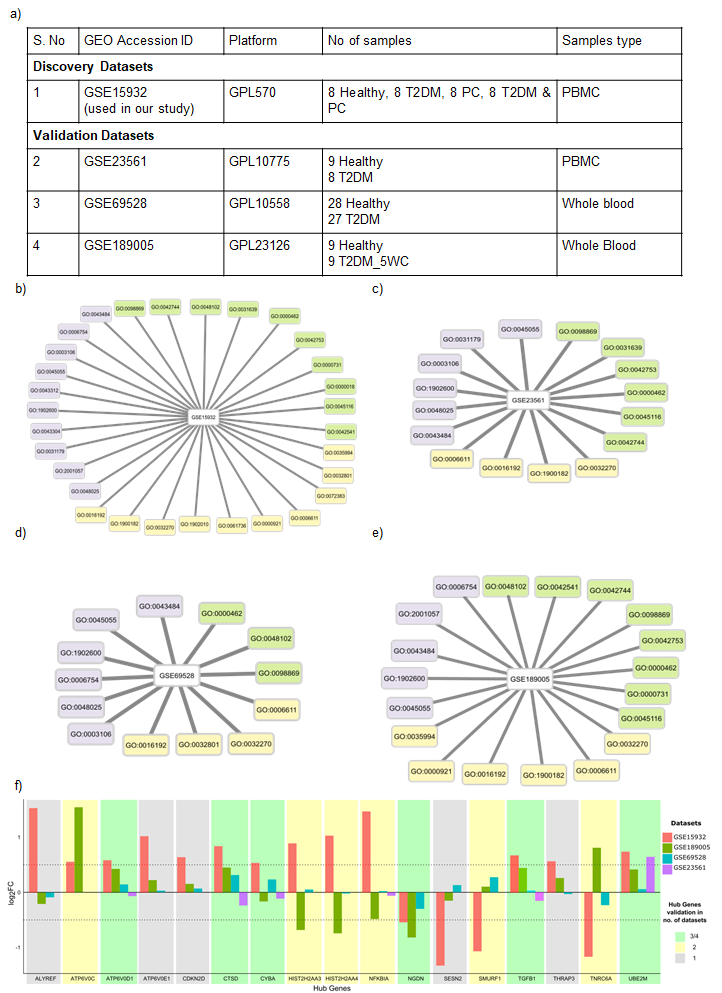


**Figure S5.** Validation of identified hub genes by comparing them to additional GEO datasets. (**a**) Table indicating expression platform, sample number, and source for the GEO datasets used for validation. (**b**) Functionally enriched gene ontology: biological processes (GO:BP) representing network for DEGs in the discovery dataset GSE15932 (used in this study) for T2DM vs. PC (yellow), T2DM vs. LC (purple) and T2DM vs. BC (green). (**c-e**) GO:BP overlaps found in the validation datasets (c) GSE23561, (d) GSE69528, and (e) GSE189005. **(f).** Expression profile for the 17 hub genes compared for discovery (GSE15932, pink bars) and validation datasets (GSE189005, green bars; GSE69528, blue bars and GSE23561, purple bars). The colored boxes on each gene denote the number of studies in which these genes were found to be significant DEG based on the raw p-values ≤0.05.


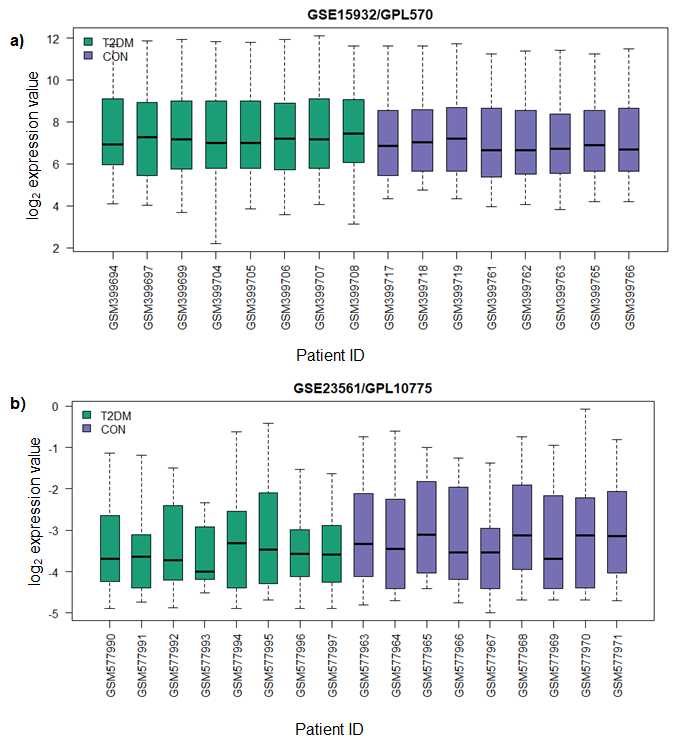


**Figure S6**: Box plot of the normalized gene expression profile for the 17 hub genes in the discovery (GSE15932, platform: GPL570) and validation (GSE23561, platform: GPL10775) dataset, where the sample source is PBMC. T2DM (green bars) and healthy control (purple bars).

**Table S1**. Top significantly enriched KEGG pathways for the shared differentially expressed genes from T2DM *vs* LC.

| **Description** | **Annotation ID** | **Count** | **FDR** | **Genes** |
| --- | --- | --- | --- | --- |
| Synaptic vesicle cycle | hsa04721 | 4 | 5.63E-02 | ATP6V0E1, ATP6V0D1, ATP6V0C, CLTB |
| Rheumatoid arthritis | hsa05323 | 4 | 5.63E-02 | ATP6V0E1, ATP6V0D1, ATP6V0C, TGFB1 |
| Collecting duct acid secretion | hsa04966 | 3 | 5.63E-02 | ATP6V0E1, ATP6V0D1, ATP6V0C |
| Phagosome | hsa04145 | 5 | 5.63E-02 | ATP6V0E1, ATP6V0D1, ATP6V0C, TUBB4B, CYBA |
| Tuberculosis | hsa05152 | 5 | 7.21E-02 | ATP6V0D1, ATP6V0C, TGFB1, NFYB, CTSD |
| Vibrio cholerae infection | hsa05110 | 3 | 7.21E-02 | ATP6V0E1, ATP6V0D1, ATP6V0C |
| Oxidative phosphorylation | hsa00190 | 4 | 9.05E-02 | ATP6V0E1, ATP6V0D1, ATP6V0C, COX5B |

**Table S2.** Differentially expressed genes (DEGs) annotated by the Cytoscape tool.

| **Comparison** | **Total DEGs** | **Annotated DEGs in Cytoscape** | **Included in Main PPI Network** | **Excluded from the PPI network** | **Additional Interactors** | **Hub** **Genes** |
| --- | --- | --- | --- | --- | --- | --- |
| T2DM **vs**PC | 46 | 42 | 16 | 26 | 30 | 7 |
| T2DM vs LC | 94 | 94 | 25 | 69 | 30 | 6 |
| T2DM vs BC | 16 | 16 | 5 | 11 | 30 | 5 |

**Table S3.** Top 15 ranked genes for T2DM &PC and T2DM *vs* LC, shorlisted on the basis of 11 different topological features from PPI network analysis. The last row in each case indicate common hub genes which were found to be top ranked as well as common for all listed topological features.

| **Topological Feature** | **Top 15 genes ranked by score (obtained from cytoHubba)** |
| --- | --- |
| **T2DM vs PC** |  |
| Degree | HIST2H2AA4, HIST2H2AA3, SMURF1, TGFB1, NFKBIA, TNRC6A, SESN2, RAD9A, TFG, ATP6V0A2, ANLN, PEX10, TRAPPC1, MYLIP, ATP6 |
| Maximal Clique Centrality | HIST2H2AA4, HIST2H2AA3, SMURF1, TGFB1, NFKBIA, TNRC6A, SESN2, RAD9A, TFG, ANLN, ATP6V0A2, PEX10, TRAPPC1, MYLIP, ATP6 |
| Density of Maximum Neighborhood Component | HIST2H2AA3, HIST2H2AA4, NFKBIA, TNRC6A, SESN2, SMURF1, TGFB1, RAD9A, TFG, ATP6V0A2, ANLN, PEX10, TRAPPC1, MYLIP, ATP6 |
| Maximum Neighborhood Component | HIST2H2AA4, HIST2H2AA3, SMURF1, TGFB1, NFKBIA, TNRC6A, SESN2, RAD9A, TFG, ATP6V0A2, ANLN, PEX10, TRAPPC1, MYLIP, ATP6 |
| Edge Percolated Component | HIST2H2AA4, HIST2H2AA3, TNRC6A, SMURF1, TGFB1, NFKBIA, RAD9A, SESN2, ANLN, PEX10, MYLIP, TFG, ATP6V0A2, ATP6, TRAPPC1 |
| Bottleneck | SESN2, TNRC6A, SMURF1, NFKBIA, RAD9A, HIST2H2AA4, HIST2H2AA3, TGFB1, ANLN, PEX10, MYLIP, TFG, ATP6V0A2, ATP6, TRAPPC1 |
| EcCentricity | TNRC6A, NFKBIA, RAD9A, SESN2, SMURF1, HIST2H2AA4, HIST2H2AA3, TGFB1, PEX10, MYLIP, KIF3A, ANLN, TFG, ATP6V0A2, ATP6 |
| Closeness | HIST2H2AA4, TNRC6A, NFKBIA, HIST2H2AA3, SMURF1, SESN2, TGFB1, RAD9A, ANLN, PEX10, MYLIP, TFG, ATP6V0A2, ATP6, TRAPPC1 |
| Radiality | TNRC6A, NFKBIA, SESN2, HIST2H2AA4, RAD9A, SMURF1, HIST2H2AA3, TGFB1, PEX10, MYLIP, ANLN, TFG, ATP6V0A2, ATP6, TRAPPC1 |
| Betweenness | SESN2, NFKBIA, SMURF1, TNRC6A, HIST2H2AA4, ANLN, HIST2H2AA3, RAD9A, TGFB1, PEX10, MYLIP, TFG, ATP6V0A2, ATP6, TRAPPC1 |
| Stress | SESN2, NFKBIA, TNRC6A, HIST2H2AA4, SMURF1, HIST2H2AA3, RAD9A, ANLN, TGFB1, PEX10, MYLIP, TFG, ATP6V0A2, ATP6, TRAPPC1 |
| *Hub genes* | *HIST2H2AA3, HIST2H2AA4, NFKBIA, SESN2, SMURF1, TGFB1, TNRC6A* |
| **T2DM vs LC** | |
| Degree | ATP6V0D1, ATP6V0C, ATP6V0E1, TGFB1, MAP2K3, CTSD, CYBA, RIOK2, ZYX, SMC5, NSMCE4A, WDR36, NFYB, H2AFJ, RRN3 |
| Maximal Clique Centrality | ATP6V0D1, ATP6V0C, ATP6V0E1, TGFB1, MAP2K3, CTSD, NFYB, CYBA, RIOK2, ZYX, SMC5, NSMCE4A, WDR36, H2AFJ, RRN3 |
| Density of Maximum Neighborhood Component | ATP6V0E1, ATP6V0D1, ATP6V0C, TGFB1, MAP2K3, CTSD, CYBA, RIOK2, ZYX, SMC5, NSMCE4A, WDR36, NFYB, H2AFJ, RRN3 |
| Maximum Neighborhood Component | ATP6V0D1, ATP6V0C, ATP6V0E1, TGFB1, MAP2K3, CTSD, CYBA, RIOK2, ZYX, SMC5, NSMCE4A, WDR36, NFYB, H2AFJ, RRN3 |
| Edge Percolated Component | TGFB1, MAP2K3, CTSD, ZYX, CYBA, NFYB, ATP6V0D1, ATP6V0C, ATP6V0E1, NSMCE4A, SMC5, H2AFJ, MPP5, UBE2M, GNB2 |
| Bottleneck | ATP6V0D1, H2AFJ, TGFB1, NFYB, ATP6V0C, ATP6V0E1, MAP2K3, CTSD, ZYX, CYBA, NSMCE4A, SMC5, WDR36, RIOK2, MPP5 |
| EcCentricity | NFYB, CTSD, UBE2M, ALDOA, ATP6V0D1, H2AFJ, TGFB1, MAP2K3, ZYX, CYBA, NSMCE4A, SMC5, MPP5, GNB2, NR1H2 |
| Closeness | ATP6V0D1, TGFB1, MAP2K3, NFYB, CTSD, H2AFJ, ZYX, ATP6V0C, CYBA, UBE2M, ALDOA, ATP6V0E1, MPP5, GNB2, NSMCE4A |
| Radiality | NFYB, TGFB1, MAP2K3, CTSD, H2AFJ, UBE2M, ALDOA, ZYX, ATP6V0D1, CYBA, MPP5, GNB2, ACADVL, NSMCE4A, SMC5 |
| Betweenness | ATP6V0D1, H2AFJ, TGFB1, MAP2K3, NFYB, ATP6V0C, ATP6V0E1, CTSD, UBE2M, ALDOA, ZYX, CYBA, MPP5, GNB2, ACADVL |
| Stress | ATP6V0D1, H2AFJ, TGFB1, MAP2K3, NFYB, ATP6V0C, ATP6V0E1, CTSD, UBE2M, ALDOA, ZYX, CYBA, MPP5, GNB2, ACADVL |
| *Common hub genes* | *ATP6V0D1, ATP6V0C, ATP6V0E1, TGFB1, CYBA, CTSD* |

**Table S4.** Description and function of identified common hub gene for T2DM *vs* PC, T2DM *vs* LC and T2DM *vs* BC.

| **Gene** | **Description** | **Function** |
| --- | --- | --- |
| **T2DM & PC** | | |
| HIST2H2AA3 | Histone cluster 2 (H2aa3) | Core component of nucleosome. Histones thereby play a central role in transcription regulation, DNA repair, DNA replication and chromosomal stability. |
| HIST2H2AA4 | Histone cluster 2 (H2aa4) | Core component of nucleosome. Histones thereby play a central role in transcription regulation, DNA repair, DNA replication and chromosomal stability. |
| NFKBIA | Nuclear factor of kappa light polypeptide gene enhancer in B-cells inhibitor, alpha | Inhibits the activity of dimeric NF-kappa-B/REL complexes by trapping REL dimers in the cytoplasm through masking of their nuclear localization signals |
| SESN2 | Hypoxia-induced gene | Functions as an intracellular leucine sensor that negatively regulates the TORC1 signaling pathway through the GATOR complex. Binding of leucine to SESN2 disrupts its interaction with GATOR2 thereby activating the TORC1 signaling pathway. This stress-inducible metabolic regulator also plays a role in protection against oxidative and genotoxic stresses |
| SMURF1 | SMAD specific E3 ubiquitin protein ligase 1 | E3 ubiquitin-protein ligase that acts as a negative regulator of BMP signaling pathway. Mediates ubiquitination and degradation of SMAD1 and SMAD5, 2 receptor-regulated SMADs specific for the BMP pathway. Plays a role in dendrite formation by melanocytes. |
| TGFB1 | Transforming growth factor, beta 1 | Multifunctional protein that controls proliferation, differentiation and other functions in many cell types. Can promote either T-helper 17 cells (Th17) or regulatory T-cells (Treg) lineage differentiation in a concentration-dependent manner. Mediates SMAD2/3 activation by inducing its phosphorylation and subsequent translocation to the nucleus. Can induce epithelial-to-mesenchymal transition (EMT) and cell migration in various cell types. |
| TNRC6A | Trinucleotide repeat-containing gene 6A protein | Plays a role in RNA-mediated gene silencing by both micro-RNAs (miRNAs) and short interfering RNAs (siRNAs). Required for miRNA-dependent repression of translation and for siRNA- dependent endonucleolytic cleavage of complementary mRNAs by argonaute family proteins. |
| **T2DM *vs* LC** | | |
| ATP6V0C | ATPase, H+ transporting, lysosomal 16kDa, V0 subunit c | Proton-conducting pore forming subunit of the membrane integral V0 complex of vacuolar ATPase. V-ATPase is responsible for acidifying a variety of intracellular compartments in eukaryotic cells. |
| ATP6V0D1 | ATPase, H+ transporting, lysosomal 38kDa, V0 subunit d1 | Subunit of the integral membrane V0 complex of vacuolar ATPase. Vacuolar ATPase is responsible for acidifying a variety of intracellular compartments in eukaryotic cells, thus providing most of the energy required for transport processes in the vacuolar system. In aerobic conditions, involved in intracellular iron homeostasis, thus triggering the activity of Fe(2+) prolyl hydroxylase (PHD) enzymes, and leading to HIF1A hydroxylation and subsequent proteasomal degradation. |
| ATP6V0E1 | ATPase, H+ transporting, lysosomal 9kDa, V0 subunit e1 | Vacuolar ATPase is responsible for acidifying a variety of intracellular compartments in eukaryotic cells |
| CTSD | Cathepsin D | Acid protease active in intracellular protein breakdown. Plays a role in APP processing following cleavage and activation by ADAM30 which leads to APP degradation. Involved in the pathogenesis of several diseases such as breast cancer and possibly Alzheimer disease. |
| CYBA | Superoxide-generating NADPH oxidase light chain subunit | Critical component of the membrane-bound oxidase of phagocytes that generates superoxide. Associates with NOX3 to form a functional NADPH oxidase constitutively generating superoxide. |
| TGFB1 | Transforming growth factor, beta 1 | Multifunctional protein that controls proliferation, differentiation and other functions in many cell types. Can promote either T-helper 17 cells (Th17) or regulatory T-cells (Treg) lineage differentiation in a concentration-dependent manner. Mediates SMAD2/3 activation by inducing its phosphorylation and subsequent translocation to the nucleus. Can induce epithelial-to-mesenchymal transition (EMT) and cell migration in various cell types. |
| **T2DM *vs* BC** | | |
| ALYREF | Transcriptional coactivator Aly/REF | It is an export adapter involved in nuclear export of spliced and unspliced mRNA. It is also involved in transcription elongation and genome stability. |
| CDKN2D | Cyclin-dependent kinase inhibitor 2D (p19, inhibits CDK4) | Interacts strongly with CDK4 and CDK6 and inhibits them; Belongs to the CDKN2 cyclin-dependent kinase inhibitor family. |
| NGDN | Centromere accumulated nuclear protein 1 | Involved in the translational repression of cytoplasmic polyadenylation element (CPE)-containing mRNAs. |
| THRAP3 | Thyroid hormone receptor-associated protein complex 150 kDa component | Involved in pre-mRNA splicing. Remains associated with spliced mRNA after splicing which probably involves interactions with the exon junction complex (EJC). Can trigger mRNA decay which seems to be independent of nonsense-mediated decay involving premature stop codons (PTC) recognition. May be involved in nuclear mRNA decay. Involved in regulation of signal-induced alternative splicing. Involved in response to DNA damage. |
| UBE2M | Ubiquitin-conjugating enzyme E2 M | It accepts the ubiquitin-like protein NEDD8 from the UBA3- NAE1 E1 complex and catalyzes its covalent attachment to other proteins. |
